# CAT-CPI: Combining CNN and transformer to learn compound image features for predicting compound-protein interactions

**DOI:** 10.3389/fmolb.2022.963912

**Published:** 2022-09-15

**Authors:** Ying Qian, Jian Wu, Qian Zhang

**Affiliations:** Shanghai Frontiers Science Center of Molecule Intelligent Syntheses, School of Computer Science and Technology, East China Normal University, Shanghai, China

**Keywords:** compound-protein interaction, drug-drug interaction, molecular image, deep learning, transformer encoder

## Abstract

Compound-protein interaction (CPI) prediction is a foundational task for drug discovery, which process is time-consuming and costly. The effectiveness of CPI prediction can be greatly improved using deep learning methods to accelerate drug development. Large number of recent research results in the field of computer vision, especially in deep learning, have proved that the position, geometry, spatial structure and other features of objects in an image can be well characterized. We propose a novel molecular image-based model named CAT-CPI (combining CNN and transformer to predict CPI) for CPI task. We use Convolution Neural Network (CNN) to learn local features of molecular images and then use transformer encoder to capture the semantic relationships of these features. To extract protein sequence feature, we propose to use a k-gram based method and obtain the semantic relationships of sub-sequences by transformer encoder. In addition, we build a Feature Relearning (FR) module to learn interaction features of compounds and proteins. We evaluated CAT-CPI on three benchmark datasets—Human, Celegans, and Davis—and the experimental results demonstrate that CAT-CPI presents competitive performance against state-of-the-art predictors. In addition, we carry out Drug-Drug Interaction (DDI) experiments to verify the strong potential of the methods based on molecular images and FR module.

## Introduction

Since developing a new drug is expensive and time-consuming, drug repurposing or repositioning is promising for drug development in the future (Manoochehri and Nourani, 2020). By contrast, repurposing an existing drug approved by the Food and Drug Administration (FDA) to obtain new drug effects saves more time and experimental funds for clinical trials (Yue and He, 2021). Therefore, silico-based methods for predicting potential Compound-protein Interactions (CPIs) are of great enhancement for drug discovery (Wu et al., 2017).

With the growth of public databases (Li et al., 2016), many computational methods have been used for the CPIs prediction. The ligand-based (Keiser et al., 2007) and docking-based (Donald, 2011) methods are the traditional computational methods. Although both methods can provide CPIs predictions, they both have obvious limitations. Ligand-based methods will not work when few binding ligands are provided for a certain target, while docking-based methods are completely dependent on the three-dimensional (3D) structure of the target (Luo et al., 2017). In recent years, machine learning based methods have been proposed to predict CPIs. Liu et al. used six typical classifiers to predict CPIs including Naive Bayes, KNN, L1-logistic, L2-logistic, support vector machine (SVM) and Random Forest (RF) (Liu et al., 2015). Yamanishi et al. proposed a supervised learning method called bipartite graph to infer interactions in drug space by synthesizing compound and protein information (Yamanishi et al., 2008). Traditional machine learning methods are based on this assumption that similar drugs may share similar targets (Lan et al., 2015). Many kernel-based methods have been proposed to follow this assumption, which essentially map various drug-drug and target-target similarity matrices (Kipf and Welling, 2016; Nascimento et al., 2016). However, the main drawback of these methods is that: they are only sensitive to small fractions of drugs which have known interactions and some datasets are of binary nature (Bagherian et al., 2021). In addition, traditional machine learning methods are difficult to perform to massive datasets to obtain great results and to understand nonlinear features.

Inspired by recent deep learning techniques, several deep learning models have been applied to drug discovery and repositioning processes that including the convolution neural network (CNN) (Öztürk et al., 2018; Wan et al., 2019) graph convolution network (GCN) (Nguyen et al., 2021), transformer (Vaswani et al., 2017; Chen et al., 2020) and the deep neural network (Gawehn et al., 2016), etc. In CPI model architecture, the process is usually divided into compound feature extraction, protein feature extraction, and classifier. The overall CPI task can be considered as a binary classification task, where the features extracted by compounds and proteins are used to determine whether there are interactions through the classifier.

One class of approach is to use deep learning to train one-dimensional compound and protein sequences. Protein information in DeepDTA (Öztürk et al., 2018) is expressed as an amino acid-based vector, where each amino acid corresponds to a unique number. Two CNN modules are then used for compound and protein sequence learning. WideDTA (Öztürk et al., 2019) is a derivative of DeepDTA, where the original drug and protein sequences are first grouped into higher-dimensional features. TransformerCPI (Chen et al., 2020) used self attention mechanism to learn the semantic relations in SMILES sequences. MolTrans (Huang et al., 2021) created a large corpus to split the original sequences, and then used transformer to encode the split sequences directly. Although, these sequence representations contain atoms and continuously learn semantic relationships between atoms, none of the sequence representations cover the spatial structure of the molecule. The loss of spatial structure information may weaken the predictive power of the model as well as the functional relevance of the learned potential space. Another family of solutions are the Graph-Based Methods which build a large heterogeneous network or create a spatial structure graph of molecular to predict CPI. The molecular graph is used as a representation of a compound molecule to learn to its spatial structure information, often using atoms as nodes and chemical bonds as edges of the graph, with the chemical valence, type and degree of the atoms as the initial node information. InterpretableDTIP (Gao et al., 2018) and CPI-GNN (Tsubaki et al., 2019; Chen et al., 2020) first convert the SMILES sequences into molecular graph with the Rdkit (Landrum, 2013) software and then use GCN for propagation and aggregation of graph node information to obtain structural features.

Extracting the molecular graph structure requires complex pre-processing of the data and multiple iterations of aggregation of the neighboring node information for each atom in the process of constructing the molecular graph. Multiple iterations may lead to the loss of information on the atomic nodes themselves.

Another recent trend is the network-based methods, which can better describe interactions between compounds and proteins by vertices and edges. Heterogeneous information networks are powerful tools for modeling the semantic information of complex data by utilizing different vertices and edges (Zhao et al., 2020). Chen et al. decomposed the heterogeneous network into multiple sub-networks and processed each sub-network separately (Chen et al., 2019). DTINet (Luo et al., 2017) learns embeddings through a network diffusion algorithm and an inductive matrix complementation strategy. Although many heterogeneous network embedding algorithms have been performed for CPI predictions, this is still challenging due to the diversity of vertex types and the diversity of relationships between vertices. In addition, heterogeneous networks only consider the correlation between drugs and targets from a macroscopic point of view, and miss thinking for the internal information of drug molecules and protein amino acids.

Over the past years, many research results in computer vision field have demonstrated that position, geometry, and spatial structure of objects in images can be well characterized. These features can greatly contribute to objects classification, detection, recognition and generation of similar objects (Zeiler and Fergus, 2014; Nguyen et al., 2016; Olah et al., 2017; Nguyen et al., 2019). In addition, the image processing field has developed rapidly in recent years, and many excellent algorithm models and technologies can be used for reference (Pouyanfar et al., 2018; Yadav and Vishwakarma, 2020). The image of the molecule clearly displays the atomic, structural, and chemical bonding information of molecules, etc. Compared with SMILES sequences, molecular graphs and the heterogeneous networks, molecular image contains quite complex information and it is reasonable to use it to represent compound.

Transformer originates from the field of natural language processing and where attention mechanism is applicable to the machine translation task (Vaswani et al., 2017). Its success has also been translated to vision tasks, including image recognition (Bello et al., 2019; Hu et al., 2019), image generation (Parmar et al., 2018; Zhang et al., 2019) and object detection (Carion et al., 2020; Hu et al., 2018). At the same time, transformer-based visual models are emerging. For example, ViT (Dosovitskiy et al., 2020) is a pure transformer model, which directly divides the images into patches and feeds them into the transformer encoder directly. PVT (Wang et al., 2021) is a pyramidal ViT, which changes the original cylinder model into a pyramidal one, greatly saving the number of computational parameters and arithmetic power. Swin Transformer (Liu et al., 2021) is a model developed by Microsoft Asia Research based on the spatial architecture of CNN networks. From this, we can find that transformer is excellent in the field of vision, i.e. image processing. Applying Transformer to the image processing field makes it possible to obtain the global information of features without increasing the depth of the network. Besides being used in the fields of computer vision and natural language processing, the self-attention mechanism of transformer is also widely used in bioinformatics. MADE (Pang et al., 2022) constructs two different encoders to learn the graph information and sequence information of the drug respectively, and then uses a feature fusion atttention-based method which integating the drug multiple dimensions features. TransPhos (Wang et al., 2022) proposes a two-stage deep learning approach and constructs three different structures of encoders for feature learning based on the attention mechanism. SDNN-PPI (Li et al., 2022) constructs three different ways of encoding protein sequences, and then uses a self-attention mechanism to further learn semantic relationships in the sequences for Protein-Protein Interaction (PPI). SAVAE-Cox (Meng et al., 2022) adopts a novel attention mechanism and takes full advantage of the adversarial transfer learning strategy, and it works for survival analysis of high-dimensional transcriptome data. Inspired by these works, we use an image-based transformer encoder to learn the information in the images of compound molecules. We use Transformer to obtain the semantic relationships between features in molecular images. Use Transformer to obtain contextual relationships between amino acids in protein sequences.

PWO-CPI (Qian et al., 2022) is our first attempt to extract features from molecular images for CPI tasks and demonstrates the potential of molecular images. In our previous work we fully explored the feasibility of molecular images as molecular feature learning. In the meanwhile, a GAN (Goodfellow et al., 2014) was constructed to demonstrate that the neural network can effectively learn the information of drug molecules contained in images. In PWO-CPI, we considered using CNN to learn feature information in molecular images by convolutional aggregation operations. However, the global information of the whole molecular image is not fully considered.

Based on the previous work, in order to further enhance the global feature learning capability, we propose a novel image-based model called CAT-CPI (combining CNN and transformer to predict CPI) which uses transformer to capture global features from images.

In this work, we first use CNN to learn the detail information in the image, and then use transformer encoder to further learn the semantic relationship of the context in global. The learning ability of molecular image is greatly enhanced by our model CAT-CPI which combining CNN and transformer. For protein feature extraction, we use a sliding window k-gram method to segment the protein sequences. The number of original amino acid species is twenty, which is insufficient for the representation of proteins. After using k-gram method, the number of amino acid combinations can be increased to $20^{k}$ .

To enhance the representation capability of the model, we propose a Feature Relearning (FR) module to learn the interaction features of compounds and proteins features. It can preserve the high-dimensional interrelationship features better, compared to the vector concatenation method. The operation of convolution in FR module can effectively capture the interrelationships between compound and protein. To validate the effectiveness of CAT and FR, we conduct experiments on three datasets and achieve the best results. The experiments were carried out in Drug-Drug Interaction (DDI) task to further verify that the CAT method is indeed effective in learning the complex information of molecular images.

## Methods

**FIGURE 1:**
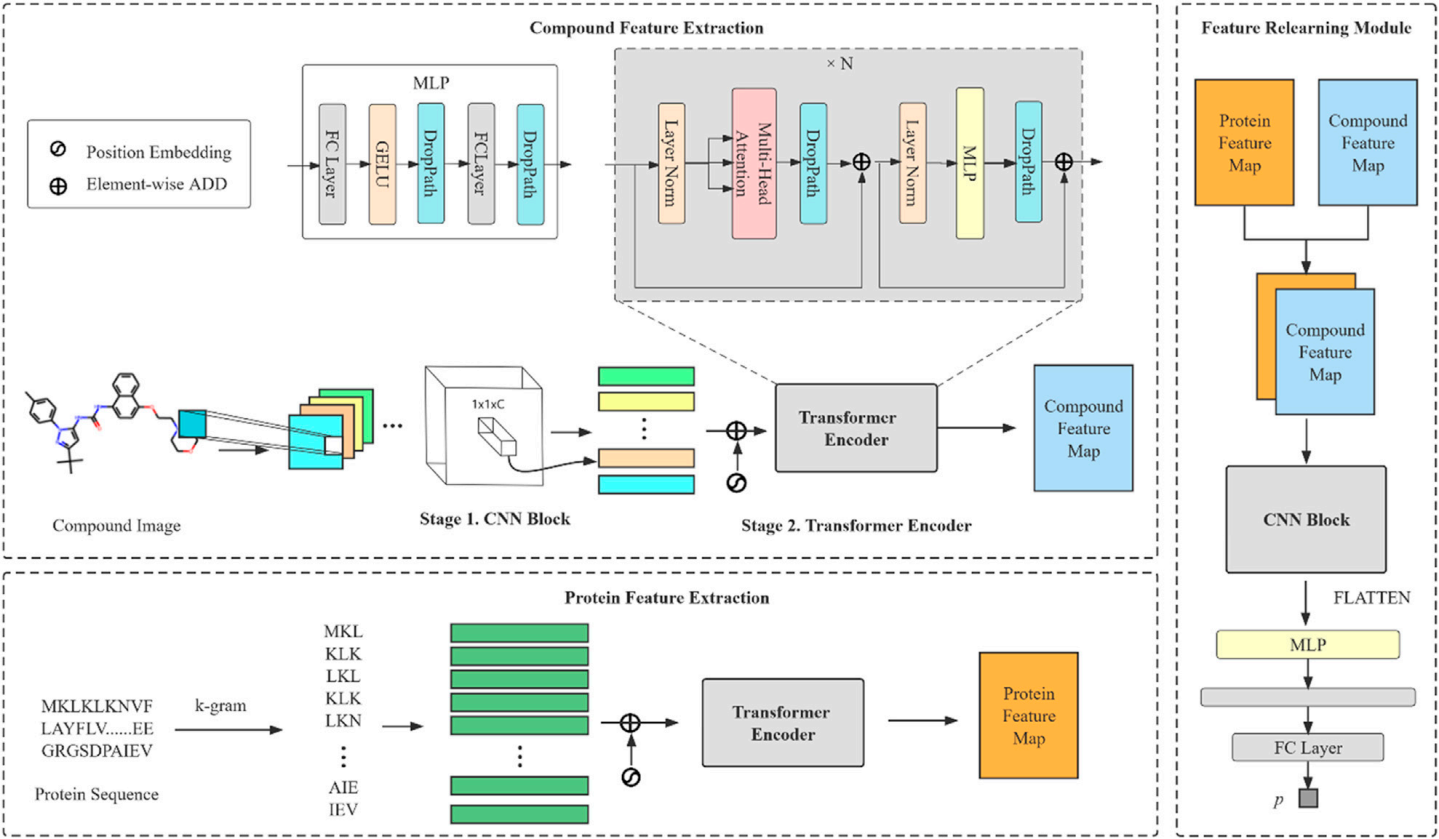
An overall architecture of the CAT-CPI. The model contains three modules: compound feature extraction, protein feature extraction and feature relearning module.

The model we proposed can be divided into three modules: compound feature extraction, protein feature extraction and FR module. The compound feature extraction is used for feature extraction of compound molecule images and the protein feature extraction is used for protein sequences extraction. FR learns the features extracted from the compound and protein feature extraction again for the final prediction. The model architecture is shown in Figure 1. Compound feature extraction is divided into two stages: CNN Block and transformer encoder. First, we construct an CNN Block to learn the local detail features of the image and conduct semantic learning by N transformer encoders. The feature map of the compound is obtained. Protein feature extraction uses k-gram method to learn the protein sequence and obtain the protein feature map. Finally, we combine both feature maps and then get the final prediction result by the FR module.

### Compound feature extraction

The compound image can be generated by Rdkit software which is denoted as $P\in R^{h\times w}$ , where h and w represent the height and width of the image respectively. This structured image is given as the input to our CNN Block. The network of our CNN Block is shown in Supplementary Figure S1, which contains convolution layers (Conv), batch normalization (Ioffe and Szegedy, 2015) layers (BatchNorm), activation layers and pool layers.

Convolution Layer. The convolutional layer is the most essential part of the CNN network, which aims to extract features from the input data. It first perceives the local features of the image, and then computes the local information by performing the convolutional aggregation operation. The process can be formulated as follows:

$$P_{out}^{i}=f\left(P*W_{conv}\right)+B_{conv}$$

where i denotes the number of layers in which network is located. *f* denotes the convolution operation and * represents dot product of matrices. $W_{conv}$ and B_conv are the parameter matrix and bias. The $p_{out}^{i}$ denotes the output of the convolution layer.

**Batch Normalization Layer.** The BatchNorm layer has the following three main roles: 1) Speed up convergence. 2) Prevent gradient exploding and gradient vanishing. 3) Prevent overfitting.

**Activation Layer.** The activation function is usually used after the convolution kernel. With the activation function, the original features are preserved and mapped, which is the key to solve the problem of nonlinearity in neural network results. In nonlinear activation layer, we use LeakyReLU (Maas et al., 2013) as the activation function, and the formula is as follows:

$$LeakyReLU\left(x\right)=\left\{ \;\begin{array}{c} \;x\;,\;if\;x\geq 0\\ \;\alpha x\;,\;otherwise \end{array}\right.$$

**Pooling Layer.** After feature extraction in the convolutional layer, the output feature maps are fed to the pooling layer for feature selection and filtering. The pooling layer contains pre-defined pooling functions whose function is to replace the result of a single point in the feature map with the feature map statistics of its neighboring regions. We choose MaxPooling as a function of the pooling layer.

After the CNN Block, we have the first step feature of the original image $W_{0}\in R^{C\times H\times W}$ , where C , H , and W imply the output channels, height and width, respectively. Then we flatten $W_{0}$ to achieve the dimensionality reduction to $W_{1}\in R^{N\times D}$ ,where N=C and D=H×W , used as the input of transformer encoder. Position embeddings are added to the $W_{1}$ to retain positional information.

Our encoder contains Layer Normalization (LN) layers, multihead self-attention (MSA), DropPath (Larsson et al., 2016) layers, MLP blocks and residual connections.

**Layer Normalization layer and Residual connections.** LN is similar to BN. The length of sequence in natural language will be inconsistent and LN can process these data well. LN is applied before every block and residual connections are after every block (Baevski and Auli, 2018; Wang et al., 2019).

**Multi-Head Self attention Layer.** MSA is an extension of self-attention. In self-attention layer, the input vector $z\in R^{N\times D}$ is transformed into three specific vectors: query vector q , key vector k and value vector v , and then these vectors are packed into different matrices Q , K and V (Vaswani et al., 2017). The computation in the self attention layer can be divided into the following steps:

Step 1: Calculate the score S of matrices Q and K: $S=Q∙K^{T}$

Step 2: Normalize the scores for gradient stability: $S_{n}=S/\sqrt{d_{k}}$

Step 3: Use softmax function to convert scores to probabilities: $P=softmax\left(S_{n}\right)$

Step 4: Obtain the weighted value matrix: SA=P∙V

The whole process can be expressed by the following equation:

$$SA\left(Q,K,V\right)=softmax\left(\frac{\left(Q\cdot K^{T}\right)}{\sqrt{d_{k}}}\right)\cdot V$$

Since the self-attention layer is insensitive to position information, it is left out of the computation process. To solve this issue, the position information is added by including the same dimensional position embedding (Shaw et al., 2018) at the time of input embedding, and the position embedding is shown by the following equation:

$$PE\left(pos,2i\right)=\sin\left(\frac{pos}{10000^{\frac{2i}{D}}}\right)$$

$$PE\left(pos,2i+1\right)=\cos\left(\frac{pos}{10000^{\frac{2i}{D}}}\right)$$

where pos implies the position of the word in sentence and i represents the current dimension of the position embedding.

In MSA layer, we run k self-attention operations, called “heads”, in parallel, and project their concatenated outputs. We set $D_{h}=D/k$ to ensure that the compute and number of parameters constant when changing k. The MSA is computed as follows:

$$MSA\left(z\right)=\left[SA_{1}\left(z\right);SA_{2}\left(z\right);\ldots ,SA_{k}\left(z\right)\right]U_{msa}$$

where $z\in R^{N\times D}$ is the input vector and $U_{msa}\in R^{k∙D_{h}\times D}$ is an linear projection matrix.

### Protein feature extraction

Proteins are characterized by their amino acid sequences. Amino acids include twenty normal types and unknown types, and unknown types are considered as one type. Therefore, the protein sequence consists of twenty-one different types amino acids. Due to the few types of amino acids and the simple representation of proteins, it becomes difficult for deep learning models to learn the features.

**FIGURE 2:**
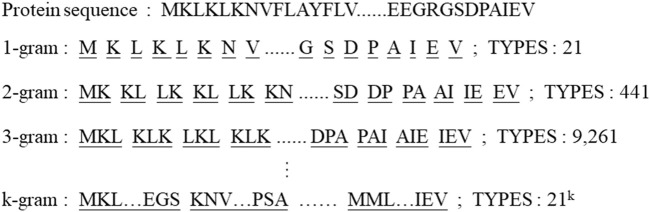
An overview of sliding window division method and types of strings of k-gram.

We use a k-gram based method to effectively solve the problem of insufficient model fit owing to the lack of amino acid types. The overview of sliding window division and number of types are shown in Figure 2. All proteins are k-gram segmented and a corpus of protein sub-sequences is built. The proteins are encoded by the numbering of the corpus library and each string is embedded according to the number of amino acid classes. The sub-sequence of a protein can be represented as $E_{i,sub}\in R^{1\times D}$ . The final protein representation is obtained by extracting N strings based on the protein length feature as $E_{i}\in R^{N\times D}$ . The chemical semantics of sub-sequences can be captured by a transformer encoder, which is the same as the one in compound feature extraction. Finally, we acquire the proteins feature map as $X_{p}\in R^{N\times D}$

### Feature relearning module and model optimization

CPI-GNN (Tsubaki et al., 2019) and GraphDTA (Nguyen et al., 2021) directly concatenate the features of compounds and proteins as the inter-action module, and then predict the results by the fully connected layer. TransformerCPI (Chen et al., 2020) feeds compound and protein features into the same transformer encoder for predicting interactions. All these methods compress the original high-dimensional features into vectors and lose the features of large number of interaction relations. Our proposed FR module can effectively retain the extracted features to obtain high dimensional features without compression. The non-linear features of the extracted features are learned by MLP and then are extracted again by CNN which has a very powerful feature aggregation capability. With FR, feature extraction of compound images and protein sequences can mostly preserve the original feature relationships. The feature map of a compound represents the spatial structure information of a drug molecule, and the feature map of a protein contains the sequence information of a protein. The interaction of the pair can be effectively extracted by the convolution operation on the stacked feature maps of compounds and proteins. The convolution kernel is convolved with molecular Information in the first layer of the feature map and then convolved with the protein sequence in the second layer in a summation operation to obtain the interrelationship between that part of the molecule and amino acids. In this way the molecular information in the image, the protein sequence information and the interaction information between atoms and amino acids are all captured.

Our FR module incudes a CNN block, a MLP block and a fully connected layer. MLP were initially recognized for their powerful feature characterization power in computer vision (Tolstikhin et al., 2021). This small CNN block is similar to CNN Block in compound feature extraction. After we get the deep representation of compound and protein, we stack the two obtained feature maps $X_{p}$ and $X_{c}$ to get $X_{out}\in R^{2\times N\times D}$ as the input of FR after MLP layer. FR module learning can be expressed as:

$$X_{out}=MLP\left(Conv_{1D}\left(Conv_{2D}\left(X_{c};X_{p}\right)\right)\right)$$

where Conv represents the operation of 1D convolution and 2D convolution, respectively. $X_{c}$ and $X_{p}$ represent the feature maps of compounds and proteins by learning, respectively and ; represents the concatenating operation of features, here spliced by channel dimension.

After relearning by MLP and CNN, we feed the final results to a fully connected layer to get the classification result $P_{n}$ . In model optimization, we choose Adam algorithm to optimize our model parameters. We set the binary cross-entropy as the loss function, as follows:

$$Loss=-\frac{1}{N}\overset{N}{\underset{n=1}{\sum }}\left(y_{n}\log\left(P_{n}\right)+\left(1-y_{n}\right)\log\left(1-P_{n}\right)\right)$$

where N is the total number of samples, and $y_{n}$ represents the true label.

## Experiments

In our experiment, we set learning rate to 0.001 and batch size to 128. The model is implemented by PyTorch 1.8. We use a server with i7 10700f, 64 GB RAM and NVIDIA 3090. The ranges of our experimental parameters settings are shown in Supplementary Table S1.

### Dataset

We choose three datasets for CPI task: namely Human (Liu et al., 2015), Celegans (Liu et al., 2015) and Davis (Davis et al., 2011). Human and Celegans are highly credible datasets with balanced positive and negative samples and are used by many researchers as experimental datasets. Davis consists of wet lab assay $K_{d}$ values among 68 drugs and 379 proteins and drug-target interaction pairs that have $K_{d}$ values < 30 units are considered positive (Davis et al., 2011). The sample distribution of these datasets is shown in Table 1. For Human and Celegans, we divided the training set, validation set and test set in the ratio of 8:1:1. We divide the dataset of Davis into 2086, 3006 and 6011 according to MolTrans (Huang et al., 2021).

**TABLE 1:** Summary of the datasets.

	Compounds	Proteins	Samples	Pos Samples
Human	1709	2043	6212	3364
Celegans	1723	1708	7511	3893
Davis	68	379	11,103	1506

In addition, we conduct DDI experiments on the Biosnap (Huang et al., 2020) dataset to verify the effectiveness of the CAT method and FR module. There are 83 041samples and 1322 drugs in Biosnap. The number of positive samples is 40,845.

### CPI experiment

We compare our model with traditional machine learning methods and deep learning methods on Human, Celegans and Davis datasets. For each experiment, we randomly run five times and then select the best model from the validation according to the AUC value. The selected models are then tested in the test set by validation. We use ROC-AUC (Area Under ROC Curve), PR-AUC (Precision Under Recall Curve), Precision, Sensitivity (Recall) and F1 scores as metrics to measure the model performance. Our methods were all randomized for 5 experiments, and the final result values were the mean and standard deviation of the multiple results. Each experiment is trained on the training set, the validation set is used to finetune the network hyperparameters, and finally the model effect is tested on the test set.

We compare CAT-CPI with traditional machine learning methods including KNN (Cover and Hart, 1967), Random Forest (RF) (Liaw and Wiener, 2002), L2 (Wright, 1995) and Support Vector Machine (SVM) (Cortes and Vapnik, 1995) on Human and Celegans, and the results are shown in Table 2. From Table 2, CAT-CPI is clearly superior to machine learning methods.

**TABLE 2:** The scores on Human dataset compared to traditional machine learning methods.

	Method	ROC-AUC	Precision	Recall
Human	KNN	0.860	0.927	0.798
	RF	0.940	0.897	0.861
	L2	0.911	0.913	0.867
	SVM	0.910	0.966	0.969
	Ours	0.986 ± 0.001	0.948 ± 0.002	0.971 ± 0.003
Celegans	KNN	0.858	0.801	0.827
	RF	0.902	0.821	0.844
	L2	0.892	0.890	0.877
	SVM	0.894	0.785	0.818
	Ours	0.992 ± 0.001	0.974 ± 0.004	0.948 ± 0.003

In addition, we make a comparison with the latest methods of deep learning model. The deep learning methods we compared are as follows:

The traditional machine learning methods are: RF, SVM, Gradient Boosting Decision Tree (GBDT) (Friedman, 2001) and Logistic Regression (LR) algorithm.

GNN-CPI (Tsubaki et al., 2019) selects molecular fingerprint information and distance matrix of molecules as the feature input of compounds, and then uses GNN network to fuse the two information for learning. We set the same hyperparameters and data model for the experiments.

DeepDTA (Öztürk et al., 2018) uses CNN for feature extraction of SMILES and protein sequences for predicting the affinity values. We add a sigmoid activation function layer at the end to turn it into a binary classification model for the DTI task and set the same hyperparameters for experimental comparison.

DeepConv-DTI (Lee et al., 2019) uses a CNN module and a global maximum pooling approach to extract local features of protein sequences and then uses a fully connected layer for feature learning on ECFP4. We obtain the same drug fingerprint ECFP4 and then set the same hyperparameters as the original paper for the experiments.

TransformerCPI (Chen et al., 2020) uses molecular sequences and distance matrices as compound feature inputs, and then constructs a Transformer encoder to learn the relationship between compound features and protein features. We construct the same sequence learning encoder and set the same hyperparameters for comparison experiments.

PWO-CPI (Qian et al., 2022) uses drug molecule image as feature sources and uses word2vec to encoder protein sequences. We build CNN module with the same process and convolutional kernels of the same size for comparison experiments.

MolTrans (Huang et al., 2021) constructs a large corpus and encoded the syllogisms, and then used a Transformer for semantic learning.

**FIGURE 3:**
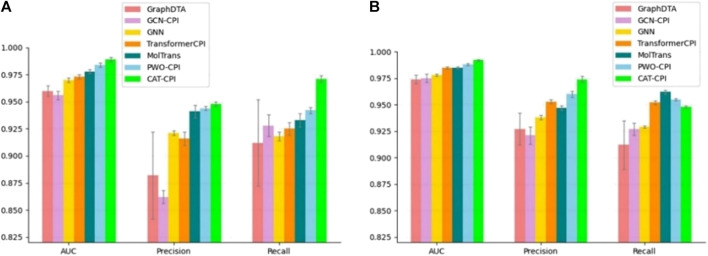
Comparison of CAT-CPI and other deep learning methods on Human **(A)** and Celegans **(B)** datasets.

The comparison results are shown in Figure 3, CAT-CPI outperforms all of these deep leaning methods in terms of AUC and Precision.

Both Human and Celegans datasets are balanced datasets. To further investigate the robustness of the model, we compare with the other methods on the Davis dataset, which is an unbalanced dataset, as shown in Table 3. It is worth noting that we can see that the results of the random forest approach to machine learning are better than most of the deep learning approaches. Because random forests then process high-dimensional data, each tree can handle unbalanced data independently of each other. Therefore, on the Davis dataset, random forest method’s performance is better than many other methods. Our main method of comparison is Moltrans, which is the state-of-the-art method on Davis dataset. The results indicate that CAT-CPI is significantly better than the other methods in all metrics.

**TABLE 3:** Comparison with other methods on Davis dataset.

Method	ROC-AUC	PR-AUC	Recall
RF	0.907	0.481	0.831
SVM	0.821	0.185	0.799
GBDT	0.836	0.271	0.755
LR	0.835 ± 0.010	0.232 ± 0.023	0.699 ± 0.051
GNN-CPI	0.842 ± 0.006	0.269 ± 0.020	0.764 ± 0.045
DeepDTA	0.880 ± 0.007	0.302 ± 0.044	0.865 ± 0.020
DeepConv-DTI	0.884 ± 0.008	0.299 ± 0.039	0.880 ± 0.024
TransformerCPI	0.841 ± 0.001	0.227 ± 0.003	0.842 ± 0.004
PWO-CPI	0.848 ± 0.001	0.278 ± 0.001	0.884 ± 0.003
MolTrans	0.907 ± 0.002	0.404 ± 0.016	0.800 ± 0.022
CAT-CPI	0.920 ± 0.001	0.481 ± 0.001	0.888 ± 0.001

**FIGURE 4:**
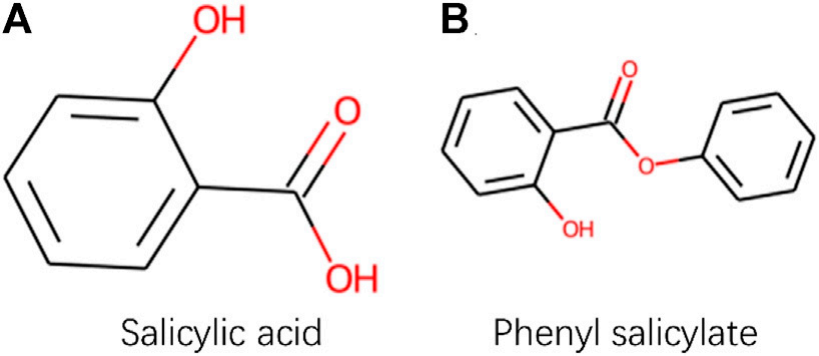
Images obtained by Rdkit based on SMILES sequences of Salicylic acid and Phenyl salicylate.

Although PWO-CPI works on the balanced datasets as shown in Figure 3 and Table 3 shows it is not as good as other methods. To find out the reason of it, we further explored the molecular images used as inputs. Figure 4 shows the molecular images of salicylic acid and phenyl salicylate obtained by Rdkit (Landrum, 2013). We can see that the two molecular images have the same size, but the sizes of the same functional group structure are different, for example, the size of the benzene ring of the salicylic acid image is larger than the size of it in the right image. Since the size of the functional group is different, the size of receptive field needed by CNN to extract same information is also different. This may lead to a weak robustness of the model.

CAT-CPI introduces to learn the global features of image molecules, which can effectively solve the problem of inconsistent receptive field size. CAT-CPI can effectively construct semantic relationships between different features through a self-attention mechanism (Vaswani et al., 2017). Thus, even if the size of functional group is different, the position of the functional group in the whole compound molecule is learned based on the semantic relationship of its context. As shown in Figure 4 and Tables 2, 3 the results on the three datasets demonstrate that CAT-CPI is quite robust.

### DDI experiments

**FIGURE 5:**
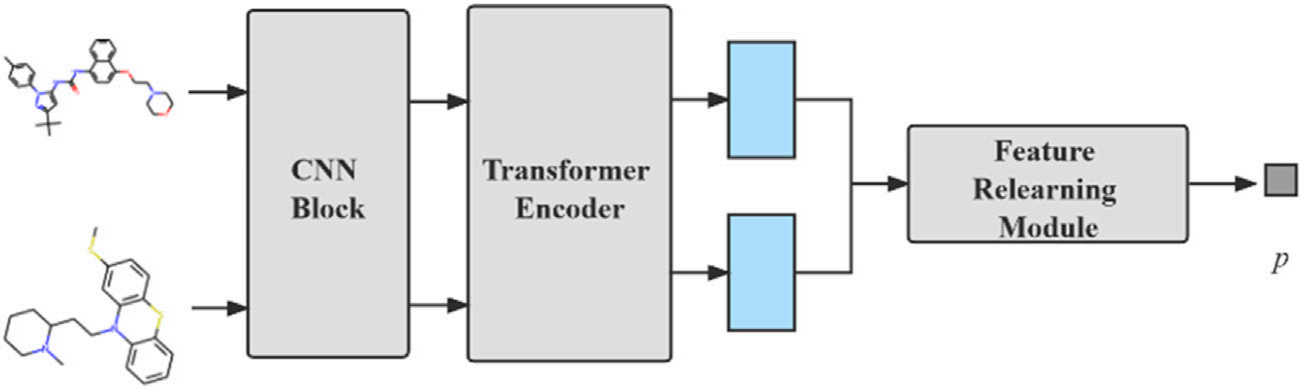
The flowchart of our DDI model. Two pictures are fed into same CNN Block and Transformer Encoder. Then the feature map is stacked to obtain the prediction results by FR module.

We use the compound feature extractor from the CAT-CPI model to deal with the DDI task to further observe the representation capability of molecular images and the feature extraction capability of our model for molecular images. The flowchart used for the DDI task is shown in Figure 5. The CNN Block and transformer encoder used here are the same as those used in the CPI task. We conduct experiment on Biosnap dataset and the methods we compared to are as follows:

1. Logistic Regression (LR) (Wright, 1995): LR with L2 regularization using representation generated from sequential pattern miningalgorithm50.
2. Nat. Prot (Vilar et al., 2014): Uses a similarity-based matrix heuristic method to build a standard model to predict DDI.
3. Mol2Vec (Jaeger et al., 2017): Applies Word2vec model to generate a dense representation of chemical structures by ECFP fingerprint.
4. MolVAE (Gómez-Bombarelli et al., 2016): Uses variable autoencoders on SMILES and generates compact representations by molecular property prediction assistance tasks.
5. DeepDDI (Ryu et al., 2018): Is a task-specific chemical similarity-based prediction model for DDI.
6. Caster (Huang et al., 2020): Is an end-to-end dictionary learning framework and incorporates a specialized representation for DDI task

The results of the DDI experiments are shown in Table 4 and other methods results are form Caster (Huang et al., 2020). The results show that our method achieves best predictive performance on DDI across all metrics. This well demonstrates the advantages of the image representation. CAT feature extraction approach and the FR module are useful to capture molecular features well. This provides a new approach to DDI tasks and has better performance than previous approaches.

**TABLE 4:** Results of DDI experiments on BIOSNAP dataset.

Method	ROC-AUC	PR-AUC	F1
LR	0.802 ± 0.001	0.779 ± 0.001	0.741 ± 0.002
Nat.Port	0.853 ± 0.001	0.848 ± 0.001	0.714 ± 0.001
Mol2Vec	0.879 ± 0.006	0.861 ± 0.005	0.798 ± 0.007
MolVAE	0.892 ± 0.009	0.877 ± 0.009	0.788 ± 0.033
DeepDDI	0.886 ± 0.007	0.871 ± 0.007	0.817 ± 0.007
CASTER	0.910 ± 0.005	0.887 ± 0.008	0.843 ± 0.005
Ours	0.960 ± 0.002	0.938 ± 0.002	0.926 ± 0.001

### Ablation study

We conduct experiments based on the size of the images in Davis dataset and the experimental results are shown in Table 5. As can be seen from Table 5, the best results are obtained for the same model parameters and computational quantities for 128 size images. Therefore, the size of the image chosen for CAT-CPI is 128.

**TABLE 5:** Experimental results of CAT-CPI on different size images.

Image_size	AUC	AUPRC	Recall
3*64*64	0.901 ± 0.001	0.371 ± 0.001	0.904 ± 0.001
3*128*128	0.920 ± 0.001	0.481 ± 0.001	0.888 ± 0.001
3*256*256	0.918 ± 0.001	0.471 ± 0.002	0.870 ± 0.001

We conduct ablation studies on a balanced dataset (Human) and an imbalanced dataset (Davis) with the following setup:

**-**CNN: We remove CNN Block in compound feature extraction from CAT-CPI. We divide the image into patches and flatten the patches and feed them into transformer encoder.
**-**Trans: We remove transformer encoder in compound feature extraction from CAT-CPI and further deepen the CNN Block.
**-**P_Trans: We remove the transformer encoder in protein feature extraction from CAT-CPI and directly use the embedding of sub-sequence as input of FR module.
**-**Word2vec: We use the word2vec model to replace our k-gram method in CAT-CPI.
**-**FR: We remove our FR module from CAT-CPI and flatten the feature map directly through the fully connected layer to obtain the result.

From Table 6, we see CNN, transformer, k-gram and RF module all contribute to the model final performance. From Table 6, we observe that when replacing the k-gram method with word2vec model to represent protein sequences, the ROC-AUC and PR-AUC have dropped a lot. From -FR results, we Find that the prediction results all show a significant decrease. Therefore, relearning of features can indeed be effective in extracting more information about interactions.

**TABLE 6:** Ablation study on Human and Davis datasets.

	Method	ROC-AUC	PR-AUC	Recall
Human	CAT-CPI	0.986 ± 0.001	0.948 ± 0.002	0.971 ± 0.003
	-CNN	0.980 ± 0.001	0.942 ± 0.004	0.942 ± 0.003
	-Trans	0.982 ± 0.001	0.939 ± 0.001	0.936 ± 0.003
	Word2vec	0.982 ± 0.001	0.925 ± 0.003	0.949 ± 0.003
	-P_Trans	0.981 ± 0.001	0.954 ± 0.003	0.936 ± 0.003
	-FR	0.966 ± 0.001	0.923 ± 0.002	0.955 ± 0.001
Davis	CAT-CPI	0.920 ± 0.001	0.481 ± 0.001-	0.888 ± 0.023
	-CNN	0.914 ± 0.003	0.473 ± 0.011	0.849 ± 0.007
	-Trans	0.912 ± 0.004	0.443 ± 0.005	0.848 ± 0.001
	Word2vec	0.908 ± 0.002	0.436 ± 0.003	0.881 ± 0.004
	-P_Trans	0.918 ± 0.002	0.478 ± 0.004	0.856 ± 0.007
	-FR	0.853 ± 0.004	0.305 ± 0.017	0.824 ± 0.022

To further explore the effectiveness of molecular images as input feature, we perform several experiments using molecular graph and compound SMILES sequence as input. We use the graph and sequence information as inputs to our model according to GNN-CPI(Tsubaki et al., 2019) and DeepDTA (Öztürk et al., 2018), and construct the model as our (GNN-CPI) and our (DeepDTA), respectively.

**Our(GNN-CPI)**: we extract the feature from molecular fingerprint and distance matrix according to GNN-CPI, and feed it into FR module, where we keep the MLP and the last fully connected layer.

**Our(DeepDTA)**: we map drug sequences to a uniform dimension similar to CAT-CPI and then use an encoder for semantic learning. Final result prediction is performed using the FR module.

We conduct experiments on the Davis dataset with the same network hyperparameters as in our model, and the experimental results are shown in Table 7. We observe that both our (GNN-CPI) and our (DeepDTA) perform better in all metrics than the original methods. In general, CAT-CPI achieves the best results in comparison with graph-based and sequence-based methods, which proves the effectiveness of molecular images as input feature.

**TABLE 7:** Network component ablation experiments on Davis dataset.

Step	ROC-AUC	PR-AUC	Recall
GNN-CPI23	0.840 ± 0.012	0.269 ± 0.020	0.696 ± 0.047
our (GNN-CPI)	0.890 ± 0.002	0.312 ± 0.003	0.816 ± 0.004
DeepDTA15	0.880 ± 0.007	0.302 ± 0.044	0.764 ± 0.045
our (DeepDTA)	0.908 ± 0.002	0.431 ± 0.002	0.845 ± 0.003
CAT-CPI	0.920 ± 0.001	0.481 ± 0.001	0.888 ± 0.001

To further validate the effect of the number of parameters and the computational quantities of the model on the experimental results. We conduct an experimental comparison of the following methods:

PWO-CPI: Only CNN is used for feature extraction of molecular images.

a) We use a transformer encoder to model the stacked feature maps.
b) We use only an MLP block and do not apply CNNs in FR.
c) We use only CNNs and do not apply MLP block in FR.
d) We use the concatenation method instead of the stacking method. We concatenate the feature maps of compounds and proteins, and then perform feature learning using 1D CNNs and an MLP block.
e) We follow the method of MolTrans to process the feature map, and perform dot product of the two feature maps. Then the features are learned by using 1D convolution and an MLP block.
f) The input image is adjusted to 64*64, and a layer of convolution is reduced when feature extraction is performed on the image.
g) The input image is adjusted to 256*256, and a layer of maximum pooling is added when feature extraction is performed on the image.

Since PWO-CPI only uses CNN for local aggregation of features, there is no operation for global feature extraction. The experimentalcomparison results of the model parameters are shown in Table 8. In order to obtain global features and interaction information, PWO-CPI performs a large number of fully connected layer calculations, which leads to increase in the number of parameters, to the extent that it is larger than the number of parameters in CAT-CPI. CAT-CPI uses CNNs with the addition of self-attention calculations compared to PWO-CPI. The number of parameters and the calculation quantities in CAT-CPI are reduced while the performance is improved significantly.

**TABLE 8:** Results of model parameters and computational quantities ablation experiments on the Davis dataset.

Method	Params (M)	FLOPs	ROC-AUC	PR-AUC	Recall
PWO-CPI	6.353	1.095G	0.835 ± 0.004	0.158 ± 0.003	0.798 ± 0.003
CAT-CPI	6.179	935.222M	0.920 ± 0.001	0.481 ± 0.001	0.888 ± 0.001
a)	2.440	486.932M	0.901 ± 0.002	0.358 ± 0.003	0.825 ± 0.002
b)	3.827	564.921M	0.854 ± 0.002	0.290 ± 0.001	0.782 ± 0.001
c)	2.113	931.159M	0.912 ± 0.001	0.441 ± 0.002	0.881 ± 0.001
d)	3.849	603.346M	0.883 ± 0.001	0.280 ± 0.002	0.860 ± 0.003
e)	5.881	586.045M	0.877 ± 0.001	0.285 ± 0.002	0.910 ± 0.002
f)	6.037	783.179M	0.901 ± 0.001	0.371 ± 0.001	0.904 ± 0.001
g)	6.179	959.602M	0.918 ± 0.001	0.471 ± 0.002	0.870 ± 0.001

Although CAT-CPI has more parameters than other methods, it obtains the best results. However, after removing the MLP block from CAT-CPI (method c), the parameter number is the lowest, but its accuracy still shows significant advantages. It proves that CNN and stacking methods in FR can greatly improve the model by adding a small number of parameters. This can also prove that the selection of each block used in our model is organized meticulously.

### Robustness experiments

To test the robustness of the CAT-CPI in the face of changes in the molecular images, we perform a random geometric transformation of all compound images on the Davis dataset, including training, validation and testing set, and re-train the model. We have conducted 4 transformation tests, including rotation, HorizontalFlip, VerticalFlip and reduction + translation. In each test, each compound is transformed randomly. Our geometric transformations include the following methods:

• Rotation: We randomly select 1/4 of the compounds to rotate 90°, 1/4 of the compounds to rotate 180°, 1/4 of the compounds to rotate 270° and keep the rest unchanged.
• HorizontalFlip: We randomly select 1/2 of the compounds to flip horizontally and keep the rest unchanged.
• VerticalFlip: We randomly select 1/2 of the compounds to flip vertically and keep the rest unchanged.
• Translation, size = (h, w): We first reduce the compound size to h*w. Then we translate the compound in four directions randomly: top-left, top-right, bottom-left, and bottom-right. The translation distance is half of the difference between the current compound size and the background size. We select 1/4 of the compounds to a top-left translation, 1/4 of the compounds to a top-right translation, 1/4 of the compounds to a bottom-left translation and the rest of the compounds to a bottom-right translation.

**FIGURE 6:**
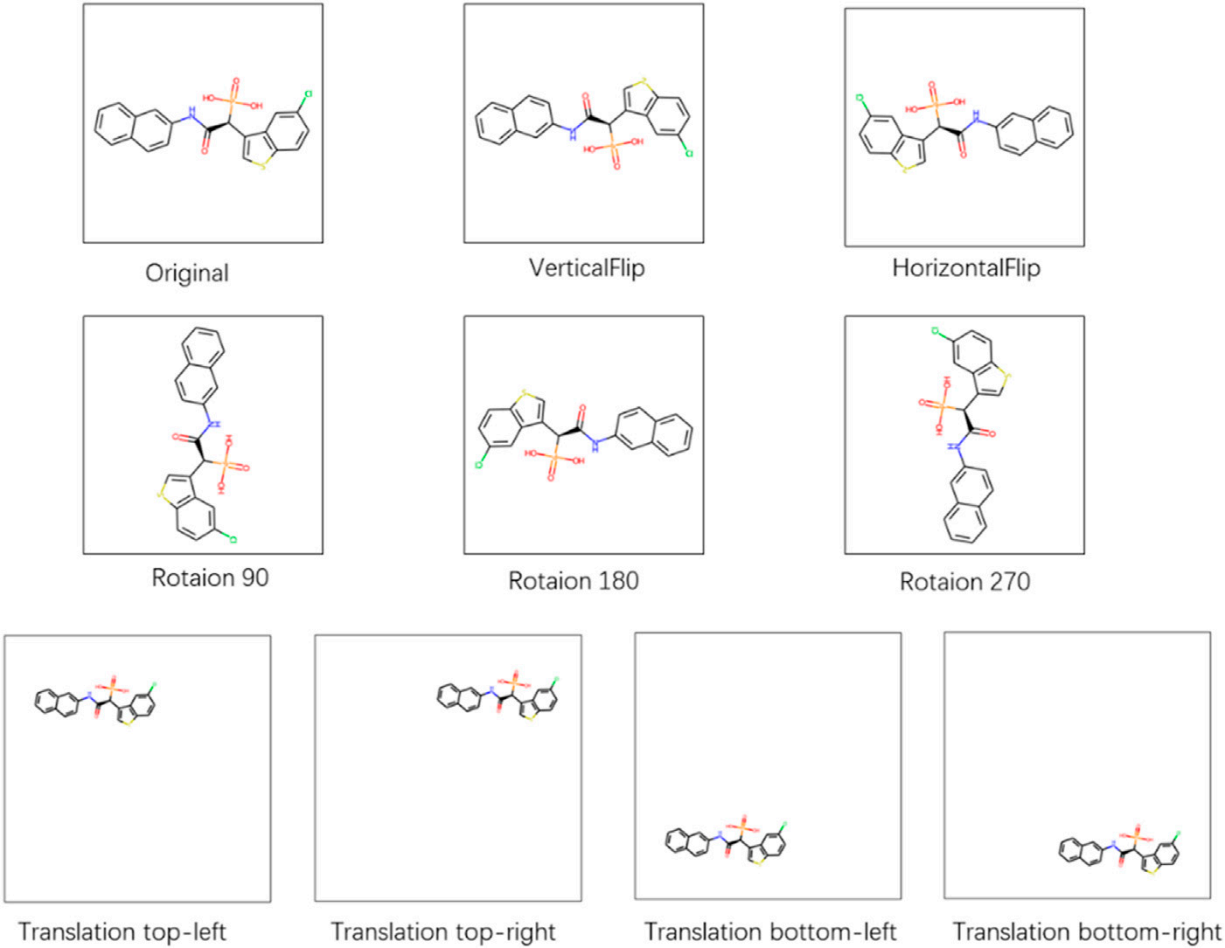
Different methods of handling compound images.

Figure 6 shows examples of these transformation methods. We do experiments using the same parameters of the model on Davis dataset. The experiments results are shown in Table 9. From Table 9, we can see that the performance of the model does decrease slightly after the geometric transformation.

**TABLE 9:** Results of the geometric transformation on the Davis dataset.

Methods	ROC-AUC	PR-AUC	Recall
Rotation	0.916 ± 0.001	0.489 ± 0.001	0.866 ± 0.001
HorizontalFlip	0.918 ± 0.001	0.488 ± 0.001	0.888 ± 0.001
VerticalFlip	0.916 ± 0.001	0.477 ± 0.001	0.867 ± 0.001
Translation, size = (96,96)	0.916 ± 0.001	0.483 ± 0.002	0.884 ± 0.002
Translation, size = (64,64)	0.911 ± 0.001	0.464 ± 0.001	0.863 ± 0.001
Translation, size = (48,48)	0.910 ± 0.001	0.423 ± 0.003	0.849 ± 0.002
Translation, size = (32,32)	0.908 ± 0.001	0.456 ± 0.001	0.860 ± 0.001

To better assess the robustness of our model, we compare with PWO-CPI which also uses the image-based method. Moreover, PWO-CPI only uses CNN to extract compound features and fuse compounds and proteins by concatenation. Therefore, PWO-CPI should not have translation invariance issue. To better understand whether the performance decrease is due to translation invariance issue or compound changes, we do the same transformation experiments using the PWO-CPI. The comparison results are shown in Table 10.

**TABLE 10:** Geometric transformation tests of PWO-CPI and CAT-CPI on the Davis dataset.

Methods	CAT-CPI (AUC = 0.920)		PWO-CPI (AUC = 0.848)	
	ROC-AUC	AUC decrease	ROC-AUC	AUC decrease
Rotation	0.916	−0.004	0.845	−0.003
HorizontalFlip	0.918	−0.002	0.844	−0.004
VerticalFlip	0.916	−0.004	0.845	−0.003
Translation, size = (96,96)	0.916	−0.004	0.845	−0.003
Translation, size = (64,64)	0.911	−0.009	0.835	−0.013
Translation, size = (48,48)	0.91	−0.010	0.834	−0.014
Translation, size = (32,32)	0.908	−0.012	0.830	−0.018

In Table 10, we can see the comparison of the performance degradation of CAT-CPI and PWO-CPI after the geometric transformation. The performance of PWO-CPI also decreases after transforming the compound images. In general, our model is able to maintain satisfactory performance in the face of image geometric transformations as well.

## Conclusion

As the feasibility and effectiveness of the image method has been confirmed in PWO-CPI, we introduce CAT-CPI, an end-to-end biological inspired molecular image-based model. Combining the local learning capability of CNN and the global representation capability of transformer to perform comprehensive representation learning of molecular images. CAT-CPI extends the word-based sequence representation of proteins to a sub-sequence representation and uses an encoder to learn the semantic relationships of sub-sequences. The FR module addresses the limitation of targeting the representation of the model without learning it completely. Comparing with other methods in CPI or DDI experiments, CAT-CPI achieves significantly improved performance on different datasets. For future works, we plan to extend it to chemical sub-image embedding and enhance features such as atomic information in molecular images for future improvement. Overall, CAT-CPI provides a novel approach to model optimization and contributes chemical biology studies with useful guidance for further.

## Data Availability

Publicly available datasets were analyzed in this study. This data can be found here: Human and Celegans can be obtained from TransformerCPI. DAVIS is available at http://staff.cs.utu.fi/∼aatapa/data/DrugTarget/; BIOSNAP is available at http://snap.stanford.edu/biodata/datasets/10002/10002-ChG-Miner.html.
