# Supplementary material for: CAT-CPI: Combining CNN and transformer to learn compound image features for predicting compound-protein interactions
Source: Front Mol Biosci. 2022 Sep 15;9:963912. doi: 10.3389/fmolb.2022.963912 (PMC9520300; doi:10.3389/fmolb.2022.963912)
Supplement: Supplementary file 1 [file DataSheet1.docx]

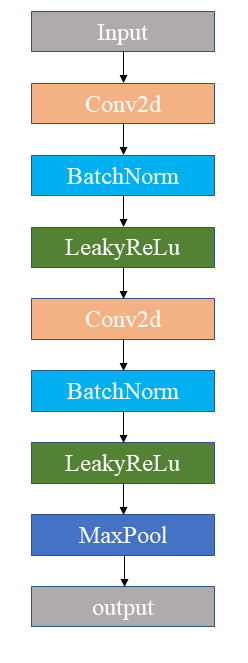


**Supplementary Figure 1.** The flowchart of CNN Block.

| Supplementary table 1. Setting of experimental hyperparameters. | |
| --- | --- |
| Hyperparameters | Values |
| Optimizer | Adam, AdamW, |
| Batch size | 64 ~ 512 |
| Learning rate | 1e-5 ~ 1e-3 |
| Learning rate decay | 0.8 ~ 0.95 |
| Weight decay | 1e-8 ~ 1e-5 |
| Decay interval | 5 ~ 20 |
| Training Epoch | 50 ~ 300 |

Supplementary table 1 shows the range of our parameter search, which is mainly referred to the following papers: ViT (Dosovitskiy et al., 2020), Swin Transformer (Liu et al., 2021), ConvNext (Liu et al., 2022), Resnet (He et al., 2016).

We use a grid search method to select the optimal parameters. Within the specified range of parameters, the parameters are adjusted sequentially in steps to find the parameter with the highest accuracy on the validation set from all the parameters.
